# Psychology’s medicalization of male baldness

**DOI:** 10.1177/13591053211024724

**Published:** 2021-06-22

**Authors:** Glen S Jankowski, Hannah Frith

**Affiliations:** 1 Leeds Beckett University, UK; 2 University of Surrey, UK

**Keywords:** bald, bias, commercial, hair loss, medicalization

## Abstract

Male baldness is physically benign though it is increasingly described as a “disease” based on claims that it is profoundly distressing. The medicalization of baldness was assessed using data extracted from a review of 37 male baldness psychosocial impact studies. Findings revealed most studies likely had commercial influences (78%), represented baldness as a disease (77%), were conducted on biased samples (68%), and advocated for baldness products/services (60%), omitting their limitations (68%). Health psychologists should challenge baldness medicalization so that men can make informed choices about what, if anything, they do with their baldness.

The balding industry is worth billions of US dollars globally (Allied Analytics LLP, 2019; Conrad et al., 2010), with hair transplants alone projected to increase in worth from 8 to 12 billion US dollars by 2026 (Allied Analytics LLP, 2019). Whilst health psychology aims to promote people’s wellbeing, it may also have facilitated the growth, actions and, ultimately, harm caused by baldness-, tobacco-, and other- commercial industries (e.g. Craig et al., 2020; Curtis, 2002; Marks and Buchanan, 2020; Ogden, 2019; Pelosi, 2019). More specifically, psychology may have facilitated the commercial balding industry through the medicalization of baldness. Baldness (also known as androgenetic alopecia or AGA) typically refers to the common occurrence of loss of hair among men that is not caused by an illness (NHS, 2018). It results in no physically harmful or life-limiting consequences (Draper, n.d.; Gonul et al., 2018; NHS, 2018; Peled, 2004; Tang et al., 2000). Nonetheless, it is medicalized^1^ where it is transformed into a disease largely on the premise that it is profoundly psychosocially distressing (British Association of Hair Restoration Surgeons, 2019; Cash, 2010). The disease status of baldness can then be used to promote “commercial interventions” (including pharmaceuticals, surgery, wigs, or other related products) as medical “treatments”. For example, the *European Dermatology Forum* guidance (Blumeyer et al., 2011; Kanti et al., 2018) provided to those who provide baldness commercial interventions (e.g. surgeons, dermatologists, etc. hereafter referred to as practitioners) refers to baldness using disease language and implicitly advocates for pharmaceutical and surgical interventions whilst also emphasizing the psychological impact of balding: “*Independent of age and gender, patients diagnosed with androgenetic alopecia undergo significant impairment in their quality of life*” (Blumeyer et al., 2011: s1).^2^ Yet this guidance relies on just two, heavily cited, studies (Alfonso et al., 2005; Cash et al., 1993) whose results do not actually support the assertion that baldness brings about significant psychological distress. Specifically Cash et al. (1993: 567) note “*balding men actively cope with their distress*” and Alfonso et al. (2005) actually found that >76% of participants never used baldness interventions, did not feel having more hair would make them more desirable to their partner and were not dissatisfied with their personal appearance.^3^ Despite claims that balding causes a significant psychosocial impact, the evidence of this is conflicting and poor quality. Specifically, studies often use non-validated measures, do not reference published norms or use a non-balding control group when assessing the impact among balding men impeding fair interpretation. Of those assessing the impact more soundly, some studies show no impact of balding (Cash et al., 1993; Passchier, 1998; Tas et al., 2018) with others showing an impact (Wang et al., 2018; Yamazaki et al., 2011).

Direct and indirect mechanisms for medicalizing baldness have been identified. For example, anti-baldness commercial campaigns indirectly medicalize baldness depicting it as something that is isolating, depressing, and stigmatized (Harvey, 2013; Moynihan et al., 2002).^4^ Baldness is also directly medicalized by using scientific, technical, and medical language to frame it as a “devastating disease” and indirectly by the promotion of pharmaceutical drugs (and sometimes hair transplant surgery) as “treatments” with no- or minimal-reference to their limitations (Conrad, 2007; Harvey, 2013; Moynihan et al., 2002). In addition, research has promoted baldness medicalization through the use of flawed methodologies and pharmaceutical influence (Jankowski, 2014).

There are several reasons why systematically assessing the medicalization of baldness through psychosocial research is important. First, the above medicalization evidence, whilst useful, is “*selective and preliminary*” (Moynihan et al., 2002: 900). Second, research like that on baldness has been “*flooded*” with conflicting findings to generate as much confusion about the state of illnesses, interventions, and benign bodily processes as possible, a systematic examination is thus necessary (Bero, 2017: 15). Third, balding men must give informed consent if electing to use commercial interventions and “*informed consent means being informed about medicalization and disease mongering*” (Moynihan et al., 2002: 900). Finally, research may be used to bolster the credentials and objectivity of baldness practitioners even if not being read directly by men themselves.

## The current study

This study sought to systematically assess male baldness medicalization by extracting data from a recent systematic review (which synthesizes the findings and methods of the psychosocial impact of baldness research; Frith and Jankowski, 2020). In contrast the current study, focuses on key mechanisms of baldness medicalization and has the following aims:

1. To assess the extent of baldness commercial conflicts of interest
2. To assess if baldnessis represented as a disease
3. To assess if intervention-orientated participants are disproportionately recruited
4. To assess if commercial interventionsare advocated
5. To assess if intervention limitations areacknowledged

## Method

### Search strategy and study selection

This paper is based on a systematic review (registered here: Frith and Jankowski, 2020; https://osf.io/uvzp9), which examined the published evidence of baldness’psychosocial impact among men.^5^ The Psychology Cross Search electronic database was systematically searched on the 9th November 2020 by combining terms for baldness, men, and psychosocial outcomes without date parameters but limited to English-language texts. The following inclusion criteria were applied: (i) the study included balding male participants; (ii) the study reported the outcomes of most of the male balding participants; and (iii) the publication reported original empirical research. Study selection was performed independently by both authors in three screening stages from titles to full text papers. Pilot screening and discussions to resolve uncertainties were also conducted.

### Data extraction

For the purposes of this paper, data regarding indicators of medicalization (conflicts of interest, baldness representation, sample biases, and intervention implications) identified through previous research (Conrad, 2007; Harvey, 2013; Jankowski, 2014; Moynihan et al., 2002) were extracted from the 37 studies in the systematic review. To provide context, other data extracted includes study characteristics (e.g. geographic location, study design), sample details (sample size, mean age, and other demographics), and a study quality score^6^ (based on a modified AXIS tool; Downes et al., (2016) where scores 0–5 = Low; 6–10 = Moderate; and 11–14 = High). Narrative results are presented below to review the studies (in line with: Liu et al., 2018) and details are summarized in Table 1.

**Table 1.** Summary of study characteristics, key results, quality assessment, and medicalization indicators (adapted from Frith and Jankowski, 2021).

Study	Location	Participant characteristics (mean age and standard deviation)^a^	Quality score	Key results^b^	Has a baldness-related commercial conflict of interest?^c^	Discloses conflict of interest?	Recruits Intervention orientated participants?	Indicates baldness is a disease?	Recommends commercial interventions?^d^	Omits treatment limitations?^d^
Alfonso et al. (2005)	Germany, France, Italy, Spain, and UK	Seven hundred and twenty nine balding men recruited via market-research or through “random digit dialling” (p. 1830; n.r.)	6	Non validated	*Merck* funding	Yes	No	Yes	Yes	Yes
Bade et al. (2016)	India	Two hundred dermatology clinic patients (*M* = 30.6, SD = 8.7)	8	Worse DLQI^e^ score	Probable^g^	Journal appears not to require disclosures	Yes	Yes	Yes	No
Budd et al. (2000)	France, Germany, Italy, and UK	Seven hundred and ninety eight balding men recruited via their households (n.r.)	6	Non comparable	*Merck* funding	Yes	No	Yes	Yes	Yes
Camacho and García-Hernández (2002)	Spain	One hundred dermatology clinic patients (n.r.)	5	Non validated	None found	Journal appears not to require disclosures	Yes	Yes	N/A	Yes
Cash (1992)	US	One hundred and three balding men referred to the study via their hairdressers	6	Non comparable	*Upjohn* funding	Yes	No	No	Yes	Yes
Cash et al. (1993)	US	Sixty dermatology clinic patients (*M* = 31.3, SD = n.r.)	7	Normal anxiety. Normal self esteem	*Upjohn* funding	Yes	Yes	No	Yes	Yes
Cash (2009)	US, UK, France, Germany, Spain, Japan, and Korea	Six hundred and fourbalding men who were interested in hair loss services/products recruited via market research (*M* = 37.1, SD = 6.6)	8	Non validated	*Merck* funding	Yes	Yes	Yes	Yes	No
Danyal et al. (2018)	Pakistan	Sixty participants likely recruited from authors’ institution	9	Worse anxiety. Normal depression. Normal self-esteem	None found	Declares none	No	Yes	Yes	Yes
DeMuro-Mercon et al. (1998)	Norway	One thousand seven hundred and sixty one balding men recruited via their households (n.r.)	6	Non comparable	*Merck* funding	Yes	No	No	N/A	N/A
Franzoi et al. (1990)	US	Fifty two balding men at a US airport (*M* = 43, SD = n.r.)	8	Worse hair-specific Skindex-29^f^ score	None found	Journal appears not to require disclosures	No	No	N/A	N/A
Ghimire (2018)	Nepal	One hundred and twenty hair transplant patients (*M* = 31.87, SD = 6.8)	7	Worse DLQI^e^ score	Probable^h^	Declares none	Yes	Yes	N/A	N/A
Girman et al. (1998)	US	Approximately 191 balding men recruited via their households (n.r.)	8	Non validated	*Merck* funding	Yes	No	Yes	N/A	N/A
Gonul et al. (2018)	Turkey	Thirty hair transplant patients (*M* = 23.47, SD = 5.79)	6	Non comparable	Probable^i^	Declares none	Yes	No	N/A	N/A
Gosselin (1984)	UK	Two hundred and four dermatology patients (*M* = 35.14, SD = 11.04)	6	Non comparable	*Tri Mil Trust*/*Institute of Trichologists* funding	Yes	Yes	No	N/A	N/A
Gupta et al (2019)	India	Two hundred dermatology patients (*M* = 32.0, SD = n.r.)	8	Worse DLQI^e^ score	Probable^j^	Declares none	Yes	Yes	Yes	Yes
Han et al. (2012)	South Korea	Nine hundred and ninety eight dermatology patients (*M* = 41.70, SD = 5.5)	6	Non comparable	Probable^k^	Journal appears not to require disclosures (does declare non-commercial funding in acknowledgements however)	Yes	Yes	Yes	Yes
Karaman et al. (2006)	Turkey	One hundred and seventy five balding men recruited from their workplaces (*M* = 34.82, SD = 9.62)	7	Non validated	Probable^l^	Journal appears not to require disclosures	n.r.	Yes	N/A	N/A
Kranz (2011)	Germany	One hundred and sixty balding men recruited from author’s university (*M* = 24.4, SD = 2.56)	10	Worse self esteem	*L’Oreal* shampoo samples used as participant incentives	Yes	No	Yes	N/A	No
Liu et al. (2019)	China	Eight hundred and seventy five hair transplant patients (*M* = 30.85, SD = n.r.)	8	Normal self esteem	Probable^m^	Declares none	Yes	Yes	Yes	Yes
Lulic et al. (2017)	Japan, South Korea, Taiwan, Mexico, and Brazil	Eight hundred and thirty five balding men who had recently received hair loss services/products (n.r.)	5	Non validated	*GlaxoSmithKline* funding	Yes	Yes	Yes	Yes	No
Maffei et al. (1994)	Italy	Sixty four dermatology patients (n.r.)	7	Non comparable	Probable^n^	Journal appears not to require disclosures	Yes	Yes	Yes	Yes
Molina-Leyva et al. (2016)	Spain	One hundred and ninety hair loss forum users (*M* = 26.3, SD = 5.4)	8	Worse Hair-specific Skindex-29^6^ score	Probable^o^	Journal appears not to require disclosures	Yes	Yes	Yes	No
Mubki et al. (2019)	Saudi Arabia	Ninety six dermatology patients (n.r.)	10	Non comparable	Probable^p^	Declares none	Yes	Yes	Yes	No
Passchier et al. (2006)	Netherlands	One hundred sixty non-hair loss dermatology patients	6	Non validated	*Merck* funding	Yes	No	Yes	Yes	Yes
Passchier et al. (1989)	Netherlands	Two hundred and one prospective or current minoxidil users (n.r.)	7	Non validated	*Upjohn* funding	Yes	Yes	Yes	N/A	No
Passchier et al. (1988)	Netherlands	Eighty five prospective or current minoxidil users (n.r.)	8	Normal general mental health. Normal anxiety	*Upjohn* funding	Yes	Yes	Yes	N/A	Yes
Rahimi-Ardabili et al. (2006)	Iran	One hundred and twenty eight dermatology patients (*M* = 25.8, SD = 4.4)	10	Normal depression	None found	Declares none	Yes	Yes	N/A	No
Russo et al. (2019)	Italy	Twenty three dermatology patients (*M* = 28.39, SD = 11.86 )	6	Normal anxiety. Worse DLQI^e^ score	Probable^q^	Declares none	Yes	Yes	Yes	N/A
Sawant et al. (2010)	India	Thirty seven dermatology patients4	7	Non comparable	Probable^r^	Declares none	Yes	Yes	N/A	N/A
Tabolli et al. (2013)	Italy	Two hundred and thirty seven dermatology patients (*M* = 31.53, SD = 10.57)	7	Worse general mental health	*Giuliani SpA* funding	Yes	Yes	Yes	Yes	N/A
Tahir et al. (2013)	Pakistan	Fifty three dermatology patients (n.r.)	7	Worse DLQI^f^ score	Probable^s^	Journal appears not to require disclosures	Yes	Yes	Yes	N/A
Tang et al. (2000)	Singapore	One hundred balding men recruited via their households (n.r.)	8	Non validated	Probable^t^	Journal appears not to require disclosures	No	No	Yes	Yes
Tas et al. (2018)	Turkey	Two hundred and eighty three dermatology patients (*M* = 23.16, SD = 6.34)	9	Normal anxiety. Normal depression. Normal self esteem	None found	Declares none	Yes	No	Yes	Yes
Van der Donk et al. (1991)	Netherlands	One hundred and sixty eight prospective or current minoxidil user (*M* = 35.0, SD = n.r.)	9	Normal anxiety	*Upjohn* funding	Yes	Yes	Yes	N/A	Yes
Wang et al. (2018)	China	Three hundred and forty dermatology patients (n.r.)	8	Worse general mental health	None found	Declares none	Yes	Yes	Yes	Yes
Wells et al. (1995)	UK	One hundred twenty two balding men recruited from UK public locations 4	8	Normal depression. Worse self esteem	None found	Journal appears not to require disclosures	No	No	N/A	N/A
Yamazaki et al. (2011)	Japan	Twenty seven prospective or current minoxidil users (*M* = 33.8, SD = n.r.)	8	Worse anxiety. Worse DLQI^e^ score	None found	Journal appears not to require disclosures	Yes	No	Yes	Yes

Notes:

a n.r. = no participant information reported (e.g. age). The exact number of balding male participants are not reported in Girman et al. (1998). Authors note however that 30% of their male sample (total sample *N* = 273) were not balding. Some of Karaman et al.’s (2006) results are incomplete and only reported for both balding men (70%, *n* = 175) and nonbalding men (30%, *n* = 77) together. Fifteen participants (4%) in Wang’s et al. (2018) study were women (total sample *N* = 355) and their results are conflated with male balding participants (*n* = 340). Finally, the following studies divided their balding male participants into subgroups: Bade into (1) older (>31 years, *n* = 122) and (2) younger (21–3, *n* = 68); Cash (1992) into (1) modest balding (*n* = 63) and (2) extensive balding (*n* = 40); Danyal into (1) mild-moderate recession (*n* = 30, *M* = 22.3, SD = –) and (2) moderate–severe recession (*n* = 30, *M* = 23.97, SD = –); Gosselin into (1) weave (*n* = 103), weave-rejected (*n* = 50), and unconcerned (didn’t try weave, *n* = 51); Passchier into (1) completed questionnaire twice (current and retrospective views on baldness; *n* = 80; age *M* = 48.0, SD = 18.2) and (2) Current questionnaire only (*n* = 80, age *M* = 50.0, SD = 18.0); Sawar into (1) young (15–26 years; *n* = 23) and older (26+ years; *n* = 14); and Wells into (1) semi bald (*n* = 60, age *M* = 36.1, SD = 13.2) and (2) severer bald (*n* = 62, *M* = 37.3, SD = 12.8).

b Key findings are reported when a study deployed a validated assessment and a nonbalding comparator sample or there were available published norms meaning balding male participant scores could be meaningfully interpreted. “Worse” indicates the study’s balding male participants had significantly worse scores on that construct compared to published norms or a comparable non balding participant sample. “Normal” indicate the study’s balding male participants had similar or better scores on the construct. Some studies used multiple assessments of the same constructs, here only the dominant finding is reported for example, Rahimi-Ardabili et al. (2006) assessed depression scores on two measures. They found normal scores on one measure and worse scores on another measure for 56% of balding male participants only. Therefore, “normal depression” is interpreted here. “Non validated” indicates study used only non-validated measures, often many single items (20+) assessing varrious experiences including dating experiences, treatment preferences, and personal confidence. We are unable to concisely summarize the results of these items here. “Non comparable” indicates study uses a measure where we cannot find any published norms to interpret scores and/or study fails to employ a sample of nonbalding participants to compare scores. Full results are synthesized in Frith and Jankowski (2021).

c *Upjohn* is a creator of a minoxidil treatment, *Merck* is the creator of *Propecia*^®^ and *Rogaine*^®^, *GlaxoSmithKline* is the creator of *Avodart*^®^, *Giuliani SpA* is the creator of *Bioscalin*^®^, and the Tri *Mil Trust/Institute of Trichologists* offer a hair loss “treatment” clinic.

d N/A = not applicable, manuscript does not discuss treatments.

e The Dermatology Quality of Life Index (DLQI; Finlay and Khan, 1994) assesses quality of life specific to dermatological conditions and is sometimes used to assess hair loss specific quality of life.

f The Hair-specific Skindex-29 (Han et al., 2012) assess quality of life specific to hair loss.

g The first author, Bade et al. (2016), of the research was a dermatologists providing baldness services at the time according to his dermatologist profile on *Practo.com* which states he has 10 years’ experience (Bade, n.d.). It appears the *Journal of Medical Science and Clinical Research* did not require disclosures.

h The author, Ghimire (2018), of the research was a hair transplant surgeon according to his dermatological clinic employer: “*[He is] one of [the] pioneers in hair transplant surgery in Nepal [who] completed more than 1000 hair transplantation cases in more than 5 years of experience in Nepal*” (Aavaran, n.d.: 12). In *J Nepal Med Assoc* the author indicates he has no conflicts of interest.

i Four of the six authors of the research (Gonul et al., 2018) list their affiliation to a dermatology clinic. Currently this clinic offers baldness interventions (Dışkapı Yıldırım Beyazıt Training and Research Hospital, 2020). The authors declare they have no conflicts of interest in the journal: *Anais Brasileiros de Dermatologia*.

j Gupta et al. (2019) was a hair transplant surgeon at the time of the research according to his dermatologist profile on *Practo.com* stating he has 11 years’ experience providing services including baldness interventions (Gupta, n.d.). The authors declare no financial support, sponsorship, or conflict of interest in the journal: *International Journal of Trichology*.

k The fourth author, Hoon Kang, of the research (Han et al., 2012) lists their affiliation to *The Catholic University of Korea*. On their webpage Kang is listed as providing “*quick treatments*” including “*hair implants*” (The Catholic University of Korea, n.d.: 5–6). The authors do not declare this, instead only acknowledging funding from the *Korean Dermatological Association* funding in the *Journal Ann Dermatol*.

l Multiple authors of the research (Karaman et al., 2006) list their affiliations to the dermatology department of *Adnan Menderes University*. It is unclear if this department offered any baldness interventions at the time. However, the first author, Göksun Karaman indicates she has privately offered baldness interventions since at least 2016 (Karaman, n.d.). It appears the journal: *International Journal of Dermatology* did not require disclosures.

m The third author, Xingdong Li, of the research (Liu et al., 2016) provided hair transplants as indicated by his stated affiliation to the “*Kafuring Hair Transplant Hospital*” and also as he is described, elsewhere, as the founder of a chain of 33 hair transplant hospitals in China (Barley Microneedle Hair Transplant Hospital, n.d.). The authors specifically note, however, that “*None of the authors has a financial interest in any of the products or devices mentioned in this manuscript.*” (p. 1441) in the journal: *Journal of Cosmetic Dermatology*.

n The third author, Rinaldi (n.d.), of the research (Maffei et al., 1994) provided baldness interventions according to his CV which states he has almost 40 years of experience of trichology-related outpatient and surgical experience. In addition, he is currently the head of research and development of *Guilliana-SpA* a pharmaceutical company that produces baldness interventions (Rinaldi, n.d.). It appears the journal: *Arch Dermatol* did not require disclosures.

o The third author, Dr. Pilar Avivar, of the research (Molina-Leyva et al., 2016) provided baldness interventions according to her employee profile noting she has provided aesthetic interventions including baldness-/trichology-related ones since July 2015 (LinkedIn, n.d.). In addition, the first author is currently employed by a clinic that provides baldness services (Virgen de las Nieves University Hospital, n.d.). The journal: *Acta Dermatovenerologica Croatica* did not appear to require disclosures.

p All authors of the research (Mubki et al., 2019) list their affiliations to dermatology clinics that “*diagnose[patients] with AGA*” (p. 31). The third author also cowrote a paper urging dermatologists to promote their cosmetic interventions including hair transplants to the wider public: “*The responses demonstrate that the Saudi Arabian public is not aware of the full scope and practice of dermatologic surgery, especially as it pertains to cosmetic procedures. Therefore, this lack of knowledge must be addressed*” (AlHargan et al., 2017: 6). The authors declare no conflicts of interest in the journal: *Egyptian Journal of Dermatology and Venereology.*

q Three of the authors of the research (Russo et al., 2019) list their affiliation to a dermatology clinic. Currently this clinic provides baldness interventions (S. Orsola-Malpighi Polyclinic, n.d.). The authors declare no conflicts of interest in the journal: *Journal of the European Academy of Dermatology and Venereology*.

r Three of the authors of the research (Sawant et al., 2010) list their affiliation to a dermatology clinic. Currently this clinic provides baldness interventions (King Edward Memorial Hospital, n.d.). The authors declare no conflicts of interest in the *International Journal of Trichology.*

s The second author, Dr. Shahbaz Aman, of the research (Tahir et al., 2013) is currently listed by a medical database (Ola Doc, n.d.) as having 28 years’ experience as a dermatologist and as providing baldness interventions. The journal: *Annals of King Edward Medical University* does not appear to require disclosures.

t Multiple authors of the research (Tang et al., 2000) list their affiliation to the *National Skin Centre*. Currently, this center provides baldness interventions (National Skin Centre, n.d.). In the author(s)’ acknowledgements section of the journal: *Singapore Medical Journal*s this probable conflict of interest is not acknowledged.

### Data sharing statement

The authors confirm that the data supporting the findings of this study are available within the article (and/or) its supplementary materials which are available at the Open Science Framework (doi: 10.17605/OSF.IO/RZP47). These include the PRISMA study protocol of the systematic review and other associated files here: https://osf.io/rzp47/?view_only=ea64dbef2787485a83e18ef0671721a7

## Results

### Disclosure of baldness commercial conflicts of interest

Seven studies reported no conflicts of interest or commercial funding (Gonul et al., 2018; Gupta et al., 2019; Mubki et al., 2019; Rahimi-Ardabili et al., 2006; Russo et al., 2019; Sawant et al., 2010; Tas et al., 2018), 12 did not disclose (Bade et al., 2016; Camacho and García-Hernández, 2002; Danyal et al., 2018; Franzoi et al., 1990; Ghimire, 2018; Karaman et al., 2006; Maffei et al., 1994; Molina-Leyva et al., 2016; Tahir et al., 2013; Tang et al., 2000; Wells et al., 1995; Yamazaki et al., 2011), and the remaining 18 indicated funding or some other potential conflict of interest.

As not all journals required these disclosures, closer inspection of the funding source, the author’s profiles and affiliations was conducted. In view of even subtle commercial influences in dermatology being (a) common (Batalla et al., 2011; Perlis et al., 2005), (b) leading to more commercially favorable results (Batalla et al., 2011; Perlis et al., 2005; Williams et al., 2006), and (c) yet often not being disclosed (Anstey, 2018; Batalla et al., 2011) and in line with best practice dermatological standards (Anstey, 2018; British Association of Dermatologists, 2016b), studies were deemed to likely have a conflict of interest if explicit evidence suggested at least one (co)author (or co(author)’s affiliated employer or the study’s commercial funders) provided baldness interventions.

### Studies that likely do not have conflict of interests

Six studies were authored or co-authored by a dermatologist but were not obviously associated with a clinic or practise providing baldness interventions (Camacho and García-Hernández, 2002; Danyal et al., 2018; Rahimi-Ardabili et al., 2006; Tas et al., 2018; Wang et al., 2018; Yamazaki et al., 2011). Two studies appeared to be authored by academics working for an academic institution (Franzoi et al., 1990; Wells et al., 1995) In total these eight studies were deemed likely baldness commercial conflict of interest free.

### Studies that likely had conflict of interests

Fifteen studies (41%) reported some commercial funding (or commercial incentives: Kranz, 2011) from companies that sold baldness products (e.g. *Merck, The Upjohn Company*, etc.; Alfonso et al., 2005; Budd et al., 2000; Cash, 1992, 2009; Cash et al., 1993; DeMuro-Mercon et al., 2000; Girman et al., 1998; Gosselin, 1984; Lulic et al., 2017; Passchier et al., 1988, 1989, 2006; Tabolli et al., 2013; van der Donk et al., 1991).

A further 14 studies (38%) very probably had at least one (co)author who directly (e.g. as a dermatologist or hair transplant surgeon) or indirectly (via their employer) provided baldness interventions at the time of the research.^7^ Seven of these studies appeared to contravene the disclosure requirements of their respective journals by not declaring these potential conflicts of interest. The journals were: *Journal of Nepal Medical Association* (Ghimire, 2018), *Anais Brasileiros de Dermatologia* (Gonul et al., 2018), *International Journal of Trichology* (Gupta et al., 2019), *Journal of Cosmetic Dermatology* (Liu et al., 2018), *Egyptian Journal of Dermatology and Venereology* (Mubki et al., 2019), *Journal of the European Academy of Dermatology and Venereology* (Russo et al., 2019), and *International Journal of Trichology* (Sawant et al., 2010). Perhaps indicating changing disclosure standards, the remaining studies appeared to publish in journals that did not require disclosures (Bade et al., 2016; Han et al., 2012; Karaman et al., 2006; Maffei et al., 1994; Molina-Leyva et al., 2016; Tahir et al., 2013; Tang et al., 2000). Perhaps the most explicit example of a hidden conflict of interest was from Liu et al. (2016: 1441) who stated in the *Journal of Cosmetic Dermatology* that “*None of the authors has a financial interest in any of the products or devices mentioned in this manuscript.*” However the study is co(authored) by Xingdong Li, a hair transplant surgeon as indicated by his stated affiliation to the “*Kafuring Hair Transplant Hospital*” (Liu et al., 2016: 1441) and also as he is the founder of a chain of 33 hair transplant hospitals in China (Barley Microneedle Hair Transplant Hospital, n.d.). Thus Li explicitly does have a financial interest in hair transplant products and indeed could gain financially from any balding men reading his (co)authored work that states “*Hair transplantation significantly elevated self-esteem level and increased satisfaction with appearance of AGA patients*” (p. 1441) and “*Cosmetic surgery is an effective way to improve patients’ appearance and psychological state*” (p. 1446). Full details of these potential conflicts of interests and their disclosure is in Table 1.

To summarize, of the 37 studies, 29 (78%) likely had explicit conflicts of interest in that their funding or incentives were provided by a company who profited from the sale of baldness products or they were (co)authored by an individual whose employer provided baldness interventions.

### Is baldness defined as a medical problem?

Five studies (14%) fully medicalized baldness referring to it as a disease (Passchier et al., 1989; Rahimi-Ardabili et al., 2006; Russo et al., 2019; Sawant et al., 2010; Tahir et al., 2013). Sixteen studies (43%) moderately medicalized baldness where they referred to it as a disorder or condition, participants as patients (even if not recruited in a medical setting) and/or described baldness as having an underlying genetic or hormonal basis^8^ (Bade et al., 2016; Budd et al., 2000; Cash, 2009; Danyal et al., 2018; Ghimire, 2018; Girman et al., 1998; Gupta et al., 2019; Han et al., 2012; Karaman et al., 2006; Kranz, 2011; Liu et al., 2018; Lulic et al., 2017; Maffei et al., 1994; Mubki et al., 2019; Tabolli et al., 2013). Six studies (16%) mildly medicalized baldness where it was not defined as a disease, condition or disorder but medical terms were used as context to baldness such as “*diagnosis*,” “*treatments*,” or “*suffering from*” (Camacho and García-Hernández, 2002; Molina-Leyva et al., 2016; Passchier et al., 1988, 2006; van der Donk et al., 1991; Wang et al., 2018).

Ten studies (27%) did not medicalize baldness (Cash, 1992; Cash et al., 1993; DeMuro-Mercon et al., 2000; Franzoi et al., 1990; Gonul et al., 2018; Gosselin, 1984; Tabolli et al., 2013; Tang et al., 2000; Tas et al., 2018; Wells et al., 1995; Yamazaki et al., 2011). Six simply did not define baldness. Four included an acknowledgement that baldness was not a disease. Three did this explicitly. Specifically, Gonul et al. (2018: 651) noted that baldness “*is biologically benign and is not a disease in the conventional sense*,” Tang et al. (2000: 204) noted “*it is not a disease in the medical sense of the word*,” and Yamazaki et al. (2011: 773) noted that baldness “*is not involved in systemic diseases*.” One final study was more suggestive about the potential non-disease status of baldness, noting “*it is a frequent complaint among adults, which mostly leads to cosmetic consequences*” (Tas et al., 2018: 185).

### Are intervention-orientated participants recruited?

One study (3%) did not report enough sampling details to determine if their participants were intervention orientated (*n* = 175; Karaman et al., 2006). Twenty-five studies (68%) representing 6,240 participants (59% of the entire sample) recruited participants who were explicitly seeking or undergoing interventions for baldness whether transplant, pharmaceutical, or cosmetic product (see Table 1). Participants from these studies were largely recruited from a dermatology clinic (e.g. Gonul et al., 2018) or through their interest in a minoxidil trial as advertised in local media (Passchier et al., 1988, 1989). Therefore, these participants were more likely to be baldness distressed than other bald men who did not take part in these studies.

Eleven studies (30%) recruited participants regardless of interest in baldness interventions forming a less biased assessment of the impact of baldness. Five of these samples were typically large, amounting to 3,604 participants (22% of the total participants) and were recruited in order to form a population representative sample (Alfonso et al., 2005; Budd et al., 2000; DeMuro-Mercon et al., 2000; Girman et al., 1998; Tang et al., 2000). The remaining six studies recruited participants through convenience sampling, and ostentatiously not because they were actively seeking or undergoing interventions (Cash, 1992; Danyal et al., 2018; Franzoi et al., 1990; Kranz, 2011; Passchier et al., 2006; Wells et al., 1995). These samples amounted to 565 participants (5% of the total sample). For example, two studies recruited participants observed by the researchers to have baldness (from a dermatology clinic but only if they attended for non-baldness related issues; Passchier et al., 2006 and from an airport; Franzoi et al., 1990). These 11 studies comprised a less biased assessment of baldness’ impact given most balding men do not seek out interventions (Alfonso et al., 2005; DeMuro-Mercon et al., 2000; Kranz, 2011; Tang et al., 2000).

### Does this psychosocial impact research recommend commercial interventions?

More than a third of the studies (*n* = 14) did not make explicit intervention recommendations. Perhaps this was because of the brevity of the papers (Camacho and García-Hernández, 2002; DeMuro-Mercon et al., 2000; Franzoi et al., 1990; Ghimire, 2018; Girman et al., 1998; Gonul et al., 2018; Gosselin, 1984; Karaman et al., 2006; Passchier et al., 1988, 1989; Rahimi-Ardabili et al., 2006; Sawant et al., 2010; van der Donk et al., 1991; Wells et al., 1995). It would be a mistake nonetheless to consider these studies free of intervention implications as some were evaluation studies and reported beneficial effects of the intervention, even unduly. For example, Passchier et al. (1988) reported non-significant results as if they were significant in their abstract (which is more often read than the body of the paper) for minoxidil: “*More psychological improvement, with regard to hair problems, social discomfort, and self-esteem occurred in the minoxidil group than in the placebo group*.” (p. 441) despite the results actually showing two or all (given the low *t* value) of these three benefits were non-significant (“*the responders were more improved than the nonresponders with respect to self-esteem (t(83)* *=* *1.87, p* *<* *0.05), overall social discomfort (a trend: p* *<* *0.10) and hair problems (NS)*” (p. 444).

Of the remaining studies that recommended interventions (*n* = 23), one study was alone in advocating psychological therapy instead of cosmetic or other interventions on the basis that men who accepted their baldness showed less distress (4%; Kranz, 2011: 347): “*Responsible practitioners and dermatologists might be correct when hesitating to medically or even surgically treat balding in young men. They should rather encourage their patients to come to terms with their baldness – not least because of limited treatment options on the one side and patient’s high expectations about treatment outcomes on the other side*.”

Five studies (22%) explicitly advocated counseling or contact with a professional psychologist but only as an adjunct to commercial (cosmetic or surgical) interventions (Mubki et al., 2019; Russo et al., 2019; Tahir et al., 2013; Tas et al., 2018; Wang et al., 2018). Ten studies (43%) advocated psychological support as an adjunct to commercial interventions but not from a professional psychologist or counselor (Bade et al., 2016; Cash, 1992, 2009; Cash et al., 1993; Danyal et al., 2018; Gupta et al., 2019; Han et al., 2012; Lulic et al., 2017; Molina-Leyva et al., 2016; Tabolli et al., 2013). The psychological support recommended was typically poorly defined (e.g. “*an empathic understanding of these patients’ concerns is essential to effective management*”; Cash, 1992: 930 and “*Psychosocial measures need to be installed*”; Danyal et al., 2018: 406). One of the few more concrete suggestions was “*a brief, structured patient questionnaire*” to screen participants (Cash, 2009: 1819) however no further recommendations indicated what practitioners should do after screening balding men (e.g. whether to refuse them intervention). Other studies (e.g. Lulic et al., 2017) noted physicians should make time for discussions with men affected psychologically, usually in terms of intervention expectations and decisions (i.e. not necessarily dealing with psychosocial issues and assuming intervention is the correct approach). Furthermore, some of these studies recommended or implied commercial interventions were the routes to resolve psychological issues associated with baldness (Cash, 1992; Danyal et al., 2018; Han et al., 2012). For example: “*Cosmetic treatments may also be used as a remedy for the psychological concern arising due to baldness*” (Danyal et al., 2018: 406).

Seven studies (30%) explicitly recommended commercial interventions. This was unspecified as just “treatments” (Alfonso et al., 2005; Maffei et al., 1994; Passchier et al., 2006), surgery, drug therapy, wigs, and hair styling (Budd et al., 2000), cosmetic surgery (Liu et al., 2018), finasteride and minoxidil (Tang et al., 2000), and finasteride (Yamazaki et al., 2011). Commercial interventions were occasionally implicitly recommended: “*The present study suggests that oral finasteride improves the QOL of patients but does not necessarily alleviate their anxieties. The guidelines for the treatment of AGA should take account of the patients’ QOL*” (Yamazaki et al., 2011: 777). More often this was explicit. For example, Passchier et al. (2006: 228) noted: “*For those who have problems, a prompt communication between the man and a dermatologist should be encouraged after the discovery, as there are now effective treatment options available for baldness and the earlier the treatment, the better.*”

### Does this psychosocial impact research acknowledge intervention limitations?

Of the 25 studies that included a reference to interventions, eight (32%; Bade et al., 2016; Cash, 2009; Kranz, 2011; Lulic et al., 2017; Molina-Leyva et al., 2016; Mubki et al., 2019; Passchier et al., 1989; Rahimi-Ardabili et al., 2006) gave some space to reporting limitations (e.g. cost, potential inefficacy, or side effects). This was usually less space than intervention benefits, but it was nonetheless more than other studies. For example, in their introduction Bade et al. (2016: 12900) noted “*In many cases the results of treatment are unsatisfactory while on the other hand, people have high expectations, hence counselling is very important in these cases.*” Kranz (2011) and Mubki et al. (2019) noted the general limitations of interventions, Lulic et al. (2017) assessed the importance of “potential side effects” (unspecified) and cost of interventions on decision making, Molina-Leyva et al. (2016) reported on the sexual dysfunction side effects of finasteride, Passchier et al. (1989: 12) acknowledged a “*transient and partial libido loss*” in 9.4% of participants who had taken minoxidil and Rahimi-Ardabili et al. (2006) reported on the side effects of finasteride. Finally, Cash (2009: 1818) reported that previous research had found finasteride and minoxidil efficaciousness was only “*relatively high*.”

Three studies acknowledged intervention limitations but only for fringe or historical interventions in contrast to finasteride, minoxidil, or surgery (12%; Gupta et al., 2019; Han et al., 2012; Tang et al., 2000). For example, Tang et al. (2000: 405) noted the potential ineffectiveness and cost of interventions but only of “*herbs, hair centers. . ..[and] traditional Chinese medicine*” and instead advocated for the “*effective help available (e.g. topical minoxidil(16) or oral finasteride(17))*.” Han et al. (2012: 317) warned against “*inappropriate and unproven therapies that are available in nonmedical settings. . . Therefore, it is necessary to provide patients with correct information that is medically approved*.” Gupta et al. (2019: 150–151) acknowledged that the “*history of the treatment of AGA has more than its fair share of trichoquackery*,” but continued to advocate for commercial interventions.

Fourteen studies did not report any limitations when mentioning interventions (56%; Alfonso et al., 2005; Budd et al., 2000; Camacho and García-Hernández, 2002; Cash, 1992; Cash et al., 1993; Danyal et al., 2018; Liu et al., 2018; Maffei et al., 1994; Passchier, 1998; Passchier et al., 2006; Tas et al., 2018; van der Donk et al., 1991; Wang et al., 2018; Yamazaki et al., 2011). These studies implied commercial interventions were universally beneficial and the default, responsible, response to baldness (e.g. by assessing how much awareness men had around interventions, Budd et al., 2000) and urged future to research to explore what “*is needed into the factors affecting men’s willingness to seek treatment for baldness*” (Alfonso et al., 2005: 1835). Other studies noted only benefits of these commercial interventions: “*Consequently, an improvement in alopecia induced by medical treatment could reduce the psychopathologic reactive symptoms by restoring the body image and its interpersonal correlates*” (Maffei et al., 1994: 871) and “*Cosmetic surgery is an effective way to improve patients’ appearance and psychological state*” (Liu et al., 2018: 1446).

## Discussion

Commercial influences on health are increasingly widespread (de Lacy-Vawdon and Livingstone, 2020; Kickbusch et al., 2016) and psychology’s facilitation of this has come under recent scrutiny (Cosgrove and Wheeler, 2013; Marks and Buchanan, 2020; Ogden, 2019; Pelosi, 2019). The medicalization of baldness represents another way psychology facilitates commercial influences of health, through the claim that baldness is psychologically devastating and thus comprises a medical disorder requiring commercial interventions to remedy (Conrad, 2007; Harvey, 2013; Jankowski, 2014; Moynihan et al., 2002). This study sought to systematically explore the mechanisms through which baldness is medicalized in research assessing its psychosocial impact.

### The dominance of baldness commercial conflicts of interest

The medicalization of baldness may arise indirectly from commercial conflicts of interest where the framing of baldness, the implications, and the conclusions in research are more commercially favorable. Analyses of dermatology research (Batalla et al., 2011; Perlis et al., 2005; Williams et al., 2006) and other research (Cosgrove and Wheeler, 2013; Lundh et al., 2017) shows commercial influences are significantly related to results and recommendations that promote commercial interventions. This is supported by one baldness study’s candid admission that commercial influence means one will “*present a [less] balance[d] picture of the likely effects of treatment*” (Passchier et al., 1989: 330). Most studies had commercial conflicts of interest (*n* = 29; 78%), although 11 of these studies did not disclose, confirming previous reports that regulation (such as disclosure) is minimal or ineffective (Anstey, 2018; Batalla et al., 2011; Cosgrove and Wheeler, 2013; John et al., 2019; Stoll et al., 2020). One common commercial funder of the research is *Merck* who have created fake academic journals, have forced researchers to sign gagging clauses and have made extensive efforts to hide product side effects (Edwards, 2009; Goldacre, 2012; Levine, 2019). However, pharmaceutical bodies are not the only vested interests in this research. Notably many of the authors provide baldness services themselves (e.g. transplants). Commercial conflicts of interest pervade baldness media and forums (Cosmeticium, 2019; Harvey, 2013; Rocknroutlaw, 2019) healthcare (Fabbri et al., 2017; Goldacre, 2012) and psychology (Conrad, 2007; Cosgrove and Wheeler, 2013). The commercial funding behind most of this research compellingly demonstrates corporate determination of health, a challenge to health psychology’s aims of promoting a healthier society.

### The disease framing of baldness

Just three studies had an explicit acknowledgement that baldness was not a disease. The rest either did not define baldness or, directly, medicalized it (e.g. definitionally). The importance of the use of medicalized language is demonstrated by a recent review (Nickel et al., 2017) which found that when more medicalized or specific terms were deployed, individuals were more favorable to invasive diagnoses or interventions and rated the condition as more severe and experienced more anxiety. The disease framing of baldness is one key direct mechanism in which corporations are determining health, in this case misrepresenting baldness as a disease.

### The dominance of intervention-orientated participants

Of the studies reporting sample details, the majority (67%) recruited male participants who were already seeking baldness services or products. Whilst this may have been for pragmatic reasons, these participants may also be: *“more concerned about the impact of alopecia on their everyday life [relative to other balding men]*” as some acknowledged (Molina-Leyva et al., 2016: 46). However, research on representative samples of balding men has found between 75% and 95% have not used or sought baldness interventions previously (Alfonso et al., 2005; DeMuro-Mercon et al., 2000; Kranz, 2011; Tang et al., 2000). By using balding men who are already seeking intervention, and thus more likely to be distressed than balding men not seeking interventions (given distress in representative sample studies is relatively low; e.g. Alfonso et al., 2005; Kranz, 2011), the research indirectly medicalizes baldness by associating it with profound psychological distress requiring medical “treatments” to remedy.

### Commercial interventions might themselves induce poor quality of life

Of the studies that made an intervention recommendation (*n* = 24), 96% recommended commercial (cosmetic, pharmaceutical, or surgical) interventions. This is notable since this research is designed to assess the psychosocial impact of baldness, often on the presumption this is profound, and yet only one study recommended psychosocial interventions (Kranz, 2011). Of the studies discussing interventions (*n* = 30), just eight (27%) including any acknowledgement of the limitations inherent in these interventions (albeit briefly). The remainder of the studies discussed interventions uncritically giving all or most of the space to discussing their benefits. This biased intervention portrayal indirectly medicalizes baldness by promoting commercial interventions as life-saving baldness “treatments”. This is concerning since all interventions, including regulatory-approved ones (finasteride and minoxidil), can be inefficacious, harmful, and expensive (NICE, 2018). Surgery carries risk of infection and evidence mounts for the existence of “postfinasteride syndrome” (Diviccaro et al., 2020; Ganzer and Jacobs, 2018; Ganzer et al., 2015) which refers to the profound and irreversible sexual, mental, and unwanted appearance changes (e.g. development of male breast tissue) some men experience even after no longer taking finasteride. A concerted effort to discredit men reporting these side effects has been noted (Diviccaro et al., 2020; Ganzer et al., 2015; Rahimi-Ardabili et al., 2006).

Whilst baldness distress is overstated in research and elsewhere (Frith and Jankowski, 2021; Harvey, 2013), a minority of balding men may be significantly distressed. It is possible commercial baldness interventions, if safely administered and monitored, provide meaningful benefits to these men. Nonetheless, bald men should be offered a menu of responses, including not intervening, when considerig their baldness. In addition, it is important that robust psychological work which seeks to understand, and alleviate, this distress is available. As Peled (2004: 1) notes, a medical professional who himself is bald: “*Certainly, the safest and least invasive way to address the psychosocial impact of baldness is through psychosocial counselling.*” This might include psychosocial interventions to promote appearance acceptance (e.g. via FaceIt@Home, Bessell et al., 2012, https://www.faceitonline.org.uk/).

It is possible that medicalization of baldness itself causes distress rather than baldness per se. Kranz (2011) found balding men who did not seek commercial interventions for their baldness were statistically less distressed than those who did. The harmful effects of some interventions, notably finasteride, may induce distress among balding men that was not present before, directly through side effects and indirectly via the way they are promoted (by portraying baldness as an isolating and suicide-inducing condition tying into a wider ‘appearance potent’ culture that includes the valorization of a full head of hair; Harvey, 2013; Jankowski et al., 2014). In other words, distress may occur because of this culture rather than baldness itself and thus, notably, not be alleviated by baldness products or services but rather wider, cultural, change. Other tentative support that medicalization promotes baldness distress comes from the acknowledgment from cosmetic and baldness professionals (British Association of Plastic Reconstructive and Aesthetic Surgeons, n.d.) that some young men are becoming distressed from balding, that they do not have, due to aggressive social marketing of commercial interventions, and that balding men may experience less distress at the moment of discovering their baldness than later on (e.g. Alfonso et al., 2005) potentially because they are not yet exposed to pervasive baldness medicalization.

## Limitations

The data reviewed was based on the published literature. Medicalization occurs outside of this (not least in unpublished and published information sources such as gray literature, anti-baldness product reviews, social media, etc). For example, Dorfman et al. (2018) found that the majority of plastic surgery social media posts by cosmetic surgeons were explicitly self-promotional (67%) rather than educational (33%) and the regulation of this is minimal (British Association of Hair Restoration Surgeons, n.d.). The assessment of medicalization should seek to document and challenge this. Further we have not tracked medicalization in relation to the development and release of baldness products (such as the development of finasteride, hair transplants, etc.) through research, which is the research balding men are more likely to access (Harvey, 2013).

## Conclusion

The results of this review show systematically that the research assessing the psychosocial impact of balding on men is largely conducted by those with commercial vested interests in baldness products and services and this research medicalizes baldness by defining it as a disease, via selective sampling of “intervention-orientated” men, and through the implicit and explicit advocacy of cosmetic and pharmaceutical products/services. Such medicalization, through psychosocial research, often by psychology authors, must be challenged if balding men are to provide informed consent in responding to their baldness.

## Research Data

sj-gsheet-1-hpq-10.1177_13591053211024724 – for Psychology’s medicalization of male baldness Click here for additional data file. sj-gsheet-1-hpq-10.1177_13591053211024724 for Psychology’s medicalization of male baldness by Glen S Jankowski and Hannah Frith in Journal of Health Psychology This article is distributed under the terms of the Creative Commons Attribution 4.0 License ( https://creativecommons.org/licenses/by/4.0/) which permits any use, reproduction and distribution of the work without further permission provided the original work is attributed as specified on the SAGE and Open Access pages ( https://us.sagepub.com/en-us/nam/open-access-at-sage).

sj-pdf-2-hpq-10.1177_13591053211024724 – Supplemental material for Psychology’s medicalization of male baldness Click here for additional data file. Supplemental material, sj-pdf-2-hpq-10.1177_13591053211024724 for Psychology’s medicalization of male baldness by Glen S Jankowski and Hannah Frith in Journal of Health Psychology

sj-txt-3-hpq-10.1177_13591053211024724 – Supplemental material for Psychology’s medicalization of male baldness Click here for additional data file. Supplemental material, sj-txt-3-hpq-10.1177_13591053211024724 for Psychology’s medicalization of male baldness by Glen S Jankowski and Hannah Frith in Journal of Health Psychology

## Notes

1. This refers to the “*transformation of ordinary processes of life into medical phenomena [through]the invisible and unregulated attempts to change public perceptions about health and illness to widen markets*” (Moynihan et al., 2002: 898–900). Medicalization was first identified in the 1970s by sociologists and disability rights activists (Conrad, 2007; Zola, 1972). Key examples of medicalization include mental disorders that have limited evidence bases (Cosgrove and Wheeler, 2013), non-complicated childbirth and appearance “defects” (Conrad, 2007). Medicalization has been criticized for being poorly specified and for pathologizing expansions in medicine as inherently harmful; criticisms that are addressed by Conrad et al. (2010) and Hofmann (2016).

2. Similar, albeit qualified, statements are made in other guidance. For example, the patient information leaflet guidance from the British Association of Dermatologists (2016a: 1) notes “*Hair loss may cause significant psychological difficulties*” and guidance from the Asian Consensus Committee (Lee et al., 2013: 1029) notes: “*Androgenetic alopecia is often associated with a poor self-image and low self-respect*.” Neither statement cites evidence.

3. Alfonso et al.’s (2005) findings are further misrepresented in the guidance by noting that: “*Men who treated their baldness successfully reported psychosocial benefits with improvements for self-esteem and personal attractiveness*” (Kanti et al., 2018: 2). This fails to note that 38%–80% of Alfonso et al.’s (2005) participants did not “*treat baldness successfully*”. Even among those who did, many (37%–62%) did not report psychosocial benefits.

4. This depiction is bolstered by research on balding stigmatization; some of which supports the idea that baldness is associated with some negative connotations (such as aging), while some show neutral or positive connotations (for a review see: Henss, 2001). This campaign and research promotes the idea baldness needs to be changed rather than stigma and fails to account for research showing baldness stigma may not translate into meaningful behaviors (Sigelman et al., 1990), may be mitigated when individuating information about the balding person is available (Kranz et al., 2019) or by other aspects of appearance such as facial hair or glasses (Blaker et al., 2020; Muscarella and Cunningham, 1996).

5. Table 1 lists these key findings from the research and a fuller synthesis of findings is available in Frith and Jankowski (2021).

6. The AXIS tool is a widely used risk of bias assessment that is appropriate for cross-sectional studies. It was developed via a systematic review of existing tools, a Delphi survey, and expert consultation (Downes et al., 2016). It is open access, has accessible guidance and is flexible in allowing modifications. Key tool criteria include whether the sample size is justified, whether attrition is noted and whether basic data is adequately described. The overall quality score of each study is presented in Table 1 and full details of each criteria are provided in our supplemental file: https://docs.google.com/spreadsheets/d/1-qg34Q_ejA7xkHshS-Basb3omvcbe4Q5RUOc90-e3KA/edit?usp=sharing

7. All potential conflicts of interests involve (co)authors of the research where publicly available information indicates they or their employer currently provides baldness interventions. It is likely this was also true at the time the studies were published given (a) some studies list the employer as the authors’ affiliation (Gonul et al., 2018; Han et al., 2012; Karaman et al., 2006; Liu et al., 2018; Mubki et al., 2019; Russo et al., 2019; Sawant et al., 2010; Tang et al., 2000), (b) some profiles of the (co)authors explicitly indicate years of experience that precedes the study publication dates (Bade et al., 2016; Ghimire, 2018; Gupta et al., 2019; Maffei et al., 1994; Molina-Leyva et al., 2016; Tahir et al., 2013), and (c) most of these studies were published relatively recently (since 2010; two studies were not; Maffei et al., 1994; Tang et al., 2000).

8. In this context, research focusing on the genetic and hormonal basis of baldness can be read as medicalizing because the research fails to note—as Conrad (2002) points out—how other aspects of the body may have genetic or hormonal bases though this does not make them a disease (e.g. eye color or moderate mood fluctuations).
