# Supplementary material for: Psychology’s medicalization of male baldness
Source: J Health Psychol. 2021 Jun 22;27(9):2161–80. doi: 10.1177/13591053211024724 (PMC9353973; doi:10.1177/13591053211024724)
Supplement: sj-pdf-2-hpq-10.1177_13591053211024724 – Supplemental material for Psychology’s medicalization of male baldness [file sj-pdf-2-hpq-10.1177_13591053211024724.pdf]

Data from Jankowski & Frith (in prep)

Principal Investigator: Glen Jankowski, PI, g.jankowski@leedsbeckett.ac.uk

Replications:
Data from the 37 published studies can be extracted using the criteria outlined in our medicalization manuscript. Per centage proportions can be calculated to replicate our findings.

File name structure: Data from Jankowski & Frith (in prep).
The base file name is composed of the name of the study and file type. THis is a google sheet.

Column headings for tabular data

1. Column A = Reference of the 37 psychosocial impact baldness study = Reference of the 37 psychosocial impact b aldness studies included extracted from Frith & Jankowski's systematic review and analysed for their medicalization .
2. Columns B - AJ = Study quality score and notes (based on modified Axis tool created by Downes et al., 2016)
3. Columns AK - AS = Medicalization indications

Codes:

1. " = as above
2. 1 = yes
3. 0 = no

Analysis:

1. Column A = None
2. Columns B - AJ = Points across the 14 criteria were totalled to gain a quality score out of 14 with higher scores in dicating higher quality.
3. Columns AK - AS = Percentages were calculated for quantifiable medicalization indicators (Columns AN, AO, A P & AR)

Updates

Any updates will take place on the google sheet which logs all edits.
